# A Smartphone Integrated Hand-Held Gap and Flush Measurement System for in Line Quality Control of Car Body Assembly

**DOI:** 10.3390/s20113300

**Published:** 2020-06-10

**Authors:** Elisa Minnetti, Paolo Chiariotti, Nicola Paone, Gisela Garcia, Helder Vicente, Luca Violini, Paolo Castellini

**Affiliations:** 1 Department of Industrial Engineering and Mathematical Sciences, Università Politecnica delle Marche, Via Brecce Bianche, 6013 Ancona, Italy; e.minnetti@pm.univpm.it (E.M.); n.paone@univpm.it (N.P.); l.violini@univpm.it (L.V.); p.castellini@univpm.it (P.C.); 2 Volkswagen Autoeuropa, 2954-024 Q.ta do Anjo, Portugal; Gisela.Garcia@Volkswagen.pt (G.G.); Helder.Vicente@Volkswagen.pt (H.V.)

**Keywords:** gap and flush measurement, laser triangulation, smartphone, industrial internet of things, in-line quality control, car-body assembly line

## Abstract

This paper presents the design and the characterization of a portable laser triangulation measurement system for measuring gap and flush in the car body assembly process. Targeting Human in the Loop (HILT) operations in the manufacturing sector, and in line with the vision of human empowerment with Industry 4.0 technologies, the instrument embeds features to ease operators’ activity and compensate possible misuse that could affect the robustness and the quality of data acquired. The device is based on a smartphone integrated with a miniaturized laser triangulation system installed in a cover. The device embodies additional sensors and control systems in order to guarantee operators’ safety (switching on and off the laser line based on specific conditions), support operators during the measurement execution task, and optimize the image acquisition process for minimizing the uncertainty associated to the measurement. The smartphone performs on-board processing and allows Wi-Fi communication with the plant IT infrastructure. Compliance to Industry 4.0 requirements is guaranteed using OPC-UA (Open Platform Communications—Unified Architecture) communication protocol enabling the exchange of live data with the plant middleware. The smartphone provides also an advanced high-resolution color display and well proven and ergonomic human–machine interfaces, which have been fully exploited in the design. The paper introduces the system optical layout and then presents the algorithms implemented to realize the gap and flush measurement. The paper finally presents the calibration of the instrument and estimates its calibration uncertainty in laboratory conditions. Then it discusses how performance decays when the operator handles the instrument on a reference car body. Finally, it shows the analysis of uncertainty when the device is used on real car bodies of different colors in a production line. It is observed that the measurement uncertainty of the whole measurement chain (measurand + instrument + operator + uncontrolled environmental conditions) is larger than the instrument calibration uncertainty because the measurement process is affected by the operator and the variable conditions of the production line.

## 1. Introduction

### 1.1. The Industrial Context

The paper presents a measurement device that integrates a miniature laser triangulation system to a smartphone. It addresses the problem of allowing an operator working in an assembly line to take gap and flush measurements and use data locally for quality control and for improving the assembly as well as store data on the factory database through a wireless connection to the factory network, thus enabling a real Industry 4.0 implementation involving humans.

According to the Industry 4.0 paradigm, zero defect manufacturing (ZDM) is one of the pillars of digital factories. A zero-defect strategy requires measured data from the process and the product and a close integration of process and quality control [1,2,3,4].

In particular, one of the fundamental strategies to approach zero-defect manufacturing is in-line quality control on 100% of production. In multi-stage production systems, the earlier a defect is detected, the better: in fact, early identification of non-conformity, performed at a single process level on parts and sub-assemblies, prevents defects from propagating to down-stream processes. Quality control is a decision-making process aimed to asses if a part or a product complies to specifications; this is done by measuring specific characteristics and comparing them to the requirements set at the design phase. The outcome is a diagnostic judgment. In such a process, measurement uncertainty plays a fundamental role; if data are uncertain, the following diagnosis will also be uncertain [5,6]. Uncertainty of measurement is therefore a central concept in digital manufacturing. In a real production scenario, however, uncertainty of measured data can be an issue, mainly due to the time varying and harsh environmental conditions in which measurements take place.

Distributed system architectures are designed and realized in multi–stage manufacturing production lines, with the purpose to integrate process and quality control. Such schemes include smart quality control stations (QCSs); they are designed to exhibit real-time adaptive behaviors in order to keep measurement uncertainty under control even in case of variations of process/product parameters; they can implement data pre-processing to derive synthetic quality indicators, self-diagnosis, and self-calibration to maximize the confidence level of the sensors output [7,8]. Even in factories with a high level of automation, operators can play fundamental roles. In fact, for a variety of technical reasons and at the same time to keep occupational levels at an ethically and economically acceptable level, humans, are often in charge of operations, sometimes complex, including measurements. Indeed, humans in the loop (HITL) not only perform production tasks but are involved in all aspects of associated decision making, thus providing a sort of human autonomy [9]; when they perform quality control tasks, they can be considered a QCS. The roles and relevance of humans in the loop, with reference to the Industry 4.0 paradigm, is clearly discussed in Cimini et al. [10]. In particular this paper identifies the main roles of human agents, one of which is data acquisition, sensing and communication. When operators are “in the measurement loop process,” specific problems arise, mainly related to human behaviors and dexterousness, which overall have an impact on measurement uncertainty; indeed, in these conditions, measurement uncertainty depends both on the measurement instrument as well as on the operator. This raises questions like ergonomics, operator training, design of human–machine interface, etc. as well as operator-dependent measurement uncertainty. This paper describes a measurement instrument specifically designed to be operated manually and connected to the factory network, so to realize the Industry 4.0 paradigm even in presence of operations done by humans, according to the industrial Internet of Things (IIoT) approach.

The focus in this paper is on a typical automotive production process, the car body assembly; this process in all state-of-the-art factories is still done by operators. In fact, the complexity of assembly operations of doors, tailgate, hood, and lights is still hampering the use of robots in these assembly lines. Indeed, a lot of operations in the line are done simultaneously by the operator that manually assembles the car parts and iteratively measures gap and flush and then corrects the alignment until measurements fall within specifications. More specifically, tailgate and rear head-lights assembly on the car body are considered. This stage of the whole assembly line is characterized by serial assembly of the parts while the car body is slowly moving on a transport belt; assembly is performed by operators, manually, with the support of simple hand-held tools or with the assistance of payload-reduction systems. Operators mount the tailgate and the headlights according to a pre-set sequence to be performed within a time window set by the takt time of the line. The geometric alignment of parts being assembled is the most relevant requirement for both aesthetic and functional reasons; in order to check the alignment and assure the assembly is within specifications, operators perform measurement of gap and flush at several points.

When operators act as described, they simultaneously play the role of an assembly machine and of a quality control station.

### 1.2. Gap and Flush Measurement in Car Assembly Lines

**Figure 1:**
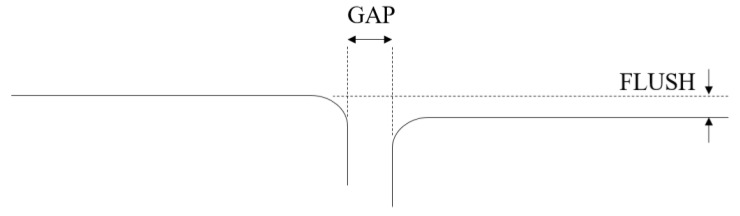
Gap and Flush definition—taken from Minnetti et al. [11].

Gap is the space between two adjacent surfaces, measured along the tangent plane to the surfaces in exam. Flush is the mismatch between two surfaces, i.e., the distance between the surfaces measured in the orthogonal direction to the tangent plane to the surfaces in exam. Figure 1 represents two adjacent parts (solid lines) and the geometrical definition of gap and flush.

Since in most assembly lines this measurement is performed manually by hand-held feeler gauges, there is no possibility to collect a continuous flow of data from the assembly process. Indeed, the only data stored and available for analysis by process engineers come from dedicated tests performed on car samples extracted from daily production.

Contrarily, the Industry 4.0 framework pushes toward continuous data flow from production line. In the car assembly context, this means acquiring gap and flush data during/along the assembly process, with the purpose of providing feedback and guidance to the operators and storing data associated to each car produced, so to move from statistical process control to 100% quality control without any increase of takt time.

The portable hand-held gap and flush measurement device described in this paper fits in the context of HITL and is specifically designed to be manually operated and support the human actively engaged in quality and process control decisions. Its non-contact nature makes it possible a fast assessment of gap and flush; moreover, it implements smart behaviors. In particular, it supports the operator during the measurement providing guidance and feedback, minimizes measurement uncertainty by adapting measurement parameters to the surface under inspection, assures a safe operation of the laser and finally is connected to the factory network for data storage and for parameters exchange. Gap and flush need to be measured in several industrial sectors, from appliances to aerospace and automotive. In car assembly, gap and flush values are significant both from an aesthetic and a functional point of view. In fact, misaligned parts are perceived as defects by the customer and, furthermore, they may cause decay in aerodynamic, aero-acoustic, and sealing performance. Gap and flush are subject to geometrical specifications, which should be checked for 100% of the products being assembled in-line.

In a typical car assembly line, there are several stages in which inspection of gap and flush takes place: in body-in-white, where parts of the frame are assembled together, the gap and flush has to be measured on rough metallic surfaces still not painted, while in later stages, the measurement takes place on metal parts painted in different colors with smooth surfaces and also involves transparent parts such as headlights. The optical characteristics of the parts vary significantly from diffusive surfaces to mirror like surfaces; the color can vary as well.

**Figure 2:**
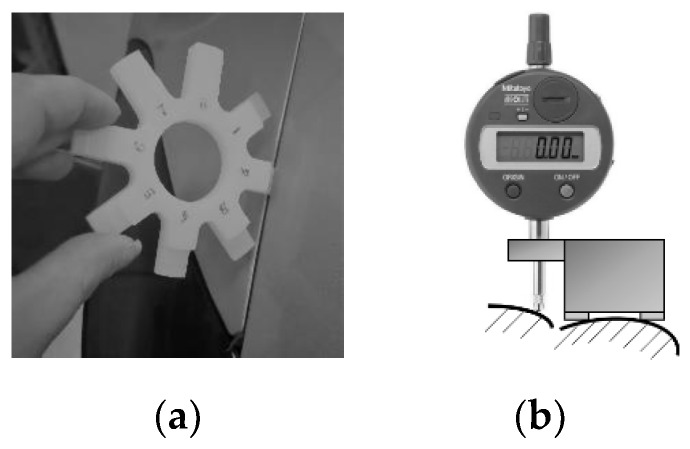
(**a**) Manual feeler gauge for gap measurement; (**b**) Dial gauge for flush measurement—taken from Minnetti et al. [11].

Conventional methods to measure gap and flush between two adjacent surfaces consist of manual feeler gauges, calipers, and dial gauges used by the operators. Figure 2a shows a picture of a toothed feeler gauge, which is used to measure gap and has a resolution of 0.5 mm, while Figure 2b shows a dial gauge to measure flush. Both systems are contact devices that provide a visual feedback to the operator. They do not allow data acquisition and storage, so there is no recorded information at the end of the inspection. In the perspective of a digital factory, this lack of stored information compromises any possible data analysis. These systems have other disadvantages. At first, these gauges involve physical contact with the target surface when measurement is done, so they can run the risk of damaging the car. Furthermore, these instruments manually operated are invasive, they can cause an increase in the size of the gap due to the interaction force between instrument and parts and therefore can bias the measurement. In general, the quality of the measurement made by these instruments is highly dependent on the positioning of the system with respect to the surfaces upon which the measurement is to be made. Furthermore, feeler gauges have large resolutions, in the order of 0.5 mm, which implies large measurement uncertainty; in addition, feeler gauges must be inserted in the gap; therefore, they can only provide a measurement smaller or equal to the actual value. Dial gauges for flush measurement can be more accurate; however, they require additional devices to be properly aligned orthogonally to the surface and their uncertainty strongly depends on operator manual skills. Finally, the process of measuring gaps and flushes with the above instruments is quite time-consuming.

### 1.3. State-of-the-Art in Optical Measurement of Gap and Flush

In the past few years, several optical systems for measurement of gap and flush have been developed for in-line measurements in automotive industry. In Kosmopoulos et al. [12], for example, a stereo cameras-based system is proposed, which has significant advantages mainly due to its colors independency. Nonetheless, the most successful approach exploits the laser line triangulation technique, a well-established optical measurement method, with several examples of laser scanners applied in-line in many industrial sectors [13,14,15]. Laser scanners can be mounted on fixed supports or on a robot manipulator; however, some hand-held solutions exist and are intended to be adopted in those assembly lines where the human in the loop is still predominant.

In Barbosa et al. [16], the metrological performance of a commercial hand-held laser instrument used in a production line of aircrafts is presented; the paper discusses repeatability and reproducibility, limited to gap measurement, and compares results to measurements acquired by a digital caliper. The paper demonstrates the competitive advantages of a laser-based measurement device over a caliper. It shows also how measurement repeatability is affected by operator handling.

Recently, 3D optical measurement systems have been integrated to smartphones. Indeed, a smartphone allows to have in a unique device a set of useful features, namely battery supply, data processing and storage capabilities, display and touch-screen interface, and wireless connectivity.

In particular, Slossberg et al. [17] propose a smartphone with the addition of a laser line projector, thus realizing a triangulation laser scanner using the camera of the smartphone. The paper presents its application to 3D reconstruction of statues, with standard uncertainty in the millimeter range but does not provide any reference to industrial applications.

In Pribanić et al. [18], a 3D structured light scanner is integrated to a smartphone; it employs an infra-red projector of a pseudorandom dot pattern. The system is demonstrated for scanning human faces; no industrial application is discussed.

There have been several issues in the automotive sector in adopting laser triangulation devices for hand-held operation related to the measurement of gap and flush. The two main issues (1) are the capability of the device to operate with the same precision and accuracy on surfaces of different optical behavior (translucent, highly reflective, etc.) and (2) the possibility to ensure that the operator can be trusted when asked to perform a measurement on those points required by the process engineers.

Recently, Minnetti et al. [11] presented the first (to our knowledge) smartphone integrated laser triangulation system for industrial application to in-line quality control of gap and flush, which was developed to try answering both the aforementioned requirements demanded by the automotive sector. This conference paper outlines the overall system characteristics, providing a general overview on the device. A number of technical reasons push towards the use of smartphones as integration platforms; in fact, a smartphone provides in a compact and ergonomic hand-held device with the following characteristics:

- computational power;
- wireless connectivity;
- operating system and programming environment;
- energy supply through its battery;
- human–machine interface, through its high-resolution large format color display.

The present paper now discusses in detail the system, hardware, and software and provides an analysis of its performance by developing a type A uncertainty analysis [19] when the device is used in operating conditions, with the scope of putting into evidence the effect of operators and uncontrolled environmental conditions (e.g., lighting in the production line) on the overall metrological performance of the system. For this scope, Section 2 and Section 3 present the system architecture, its components and the hardware and software solutions adopted on the device. Section 4 discusses the metrological performance of the instrument in laboratory conditions and in real assembly line (Volkswagen Autoeuropa—VWAE). Section 5 draws the main conclusions of the work.

## 2. Materials and Methods

### 2.1. Hand-Held Device: Hardware Design

**Figure 3:**
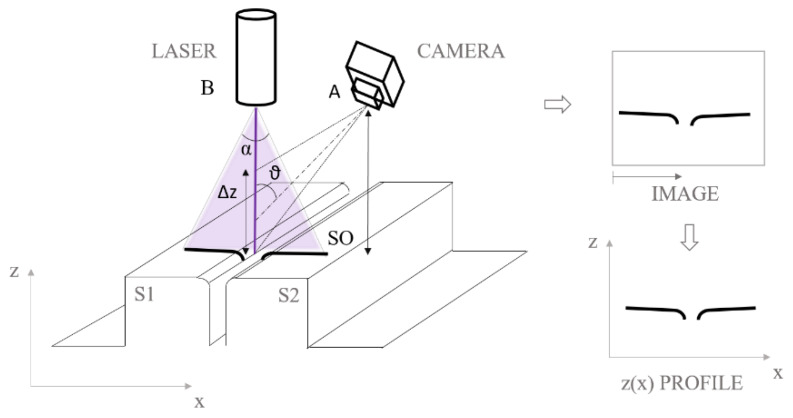
A laser triangulation system’s working principle.

Laser line triangulation is a well-known technique: a laser line is projected onto a target surface while a camera, framing the scene from a certain angle *θ*, detects light scattered by the target surface. Even if a laser scanner is capable of providing3D shape, the scope of this paper is to address gap and flush measurement, not a full 3D shape reconstruction. Figure 3 shows the laser triangulation system, implemented with the laser axis orthogonal to the surface and an inclined camera. Once the laser profile z(x) is extracted from the raw image, gap and flush values can be calculated by dedicated processing algorithms.

**Figure 4:**
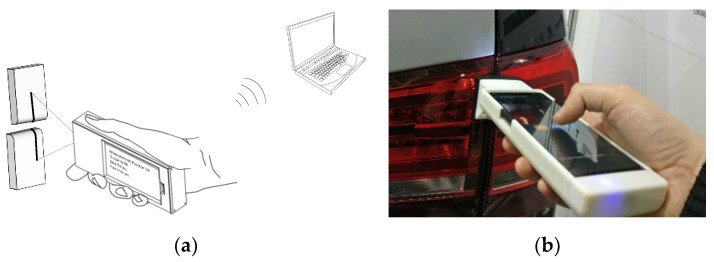
(**a**) Integration of a laser scanner to a smartphone: preliminary concept taken from the patent [18] showing device use and connectivity; (**b**) Final solution showing an operator operating the device on a car tailgate.

Laser triangulation technology is well known; however, no commercial device today offers a series of characteristics like portability, computational power, wireless network connectivity, sensing capabilities, battery supply, a human–machine interface, and the possibility to implement smart behaviors, all integrated to a smartphone. Indeed, all the former characteristics are typical of today’s smartphones. For this reason, it has been quite straightforward to scale down the optical triangulation system to insert it into a smartphone cover. Figure 4 shows a sketch of the device in the hands of an operator (the picture is taken from the patent associated to the device [20]) and a picture taken while measuring gap and flush on rear headlights.

The cover is realized in polylactide (PLA) by additive manufacturing; it hosts the smartphone and has a front hood hosting the laser triangulation system. A rubber seal covers the front part of the hood, which constitutes the measuring end of the device: this solution allows the system to take measurements with contact or non-contact to the target surface. Indeed, if the hood is put into contact with the target surface, the benefits are twofold: the hood prevents ambient light from spoiling the measurement, while the rubber seal prevents scratches on the surface.

With reference to the notation reported in Figure 3, it should be highlighted that the laser line projector is mounted to have the projected line parallel to the smartphone display. This way an operator should project the laser line orthogonally to the target surface and to the gap to be measured, as described in Figure 4. The aperture angle of the laser sheet is α = 60°. The camera (also hosted in the cover) has its optical axis forming an angle θ = 45° with respect to the laser axis. It is true that a solution with the camera inclined with respect to the laser loses sensitivity in the *z* direction if compared to the reversed solution (optical axis of the camera perpendicular to the target and optical axis of the laser inclined with respect to the camera); however, this specific design choice was adopted since it gives the best performance on surfaces ranging from optical diffusive behavior (e.g., metal parts) to highly reflective ones (e.g., chromed parts). In fact, if the solution with inclined laser was adopted, the amount of light scattered to the camera sensor from highly reflective surfaces would have been too small, thus decreasing the signal-to-noise ratio on the image and therefore increasing the uncertainty on the measurement. The system is designed to operate at a stand-off distance SO = 20 mm, over a range Δz = 15 mm.

In designing the laser triangulation system, the choice of the laser wavelength is extremely relevant. The following variables have to be considered:

1. optical properties of the target surface (scattering, diffusivity, absorption);
2. characteristics of laser, wavelength and coherence;
3. spectral sensitivity of the camera.

For what the optical properties of target surfaces are concerned, one may find the following conditions in a car assembly process:

(a) rough galvanized metal sheet not painted;
(b) smooth, polished and painted metal sheet;
(c) smooth polished and painted polymeric surfaces;
(d) smooth transparent surfaces with different color, in glass or polymers.

**Figure 5:**
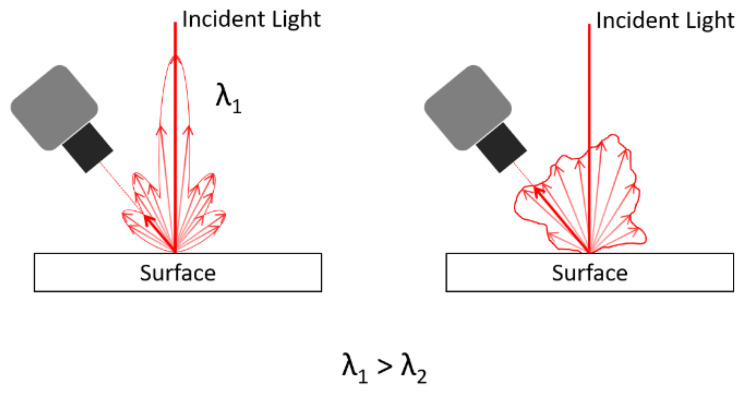
Scattering from a generic surface having roughness Ra; in a laser triangulation system, the shorter wavelength on the right provides higher signal collected by the camera.

The portable laser system addressed in this paper is meant to be used at the final assembly of the car doors, tailgate and lights, therefore it will operate on surfaces type (b), (c), and (d), i.e., smooth surfaces with different colors and eventually transparent surfaces. Surface roughness Ra strongly influences the angular distribution of scattered light. For smooth polished surfaces, such as those of our application, Ra is very small, in the same order of magnitude of the wavelength of visible light λ. Despite roughness being small, the range of Ra values that interests surface types (b), (c), and (d) is wide; hence, the choice of laser wavelength has to be made as a trade-off, i.e., as the wavelength ensuring overall high performance on the whole range. In making this choice, it should be recalled that when the ratio Ra/λ decreases, the surface tends to become a mirror, and scattered light is very directional. Figure 5 shows a qualitative sketch of the angular distribution of scattered light (different wavelengths) from rough surfaces. The shorter the wavelength, the wider the scattered light diagram. In a laser triangulation system, this is detrimental because it reduces light collected by the camera lens, thus reducing image contrast. To improve diffusive light scattering, the choice of a short wavelength is therefore mandatory in order to have an optically rougher surface (higher Ra/λ ratio). From this analysis, in order to improve angular light scattering, a violet laser source is the best candidate because of its short λ.

A second aspect to be dealt with is light reflectance at different wavelength. Spectral reflection coefficient of different surfaces depends on surface colors, coating layers, and pigments.

For what concerns painted metal, different painting colors provide different reflectance values. A spectrophotometric analysis of different painted metal surfaces was performed (not reported here): white color is characterized by the flattest and largest reflectance, in the order of 80% over the whole visible spectrum, while black is characterized by the lowest reflectance, in the order of 10% over the whole visible spectrum. Given these large differences in reflectance over the whole spectrum, it is difficult to identify the optimal wavelength for a generic painted metal surface.

**Figure 6:**
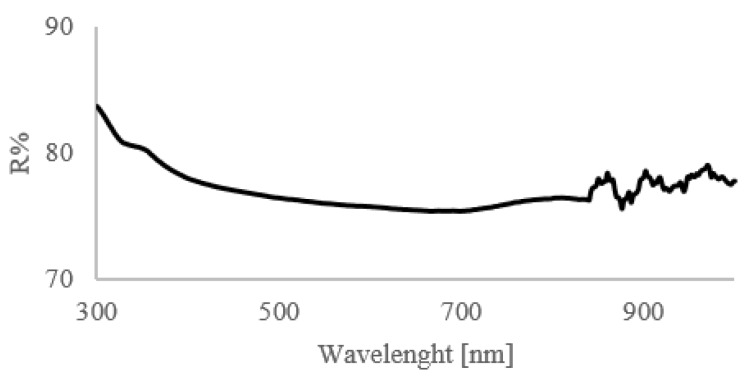
Total spectral reflectance percentage in different wavelengths for a plastic headlight sample.

Concerning headlights, their spectral reflectance depends on the color of the transparent plastic layers, on the characteristics of the functional facets that are involved in redirecting the incoming light, and on technology used for their fabrication [21]. We measured the spectral reflectance with a spectrophotometer, and it can be observed that plastic headlights present an increase in the total reflectance in the lower spectral range, i.e., the blue to violet region (Figure 6).

The final choice on laser wavelength fell on a violet laser (λ = 405 nm); in fact, this choice satisfies requirements set by angular light scattering on smooth surfaces and improves reflectance on plastic headlights while keeping a sufficient reflectance on metal surfaces painted in different colors. Moreover, laser diodes working at this wavelength are well available on the market (low-cost), as are their conditioning optical components.

A final remark concerns the choice of the laser source is as follows: low coherence lasers are to be preferred, because the resulting speckle pattern is less evident, thus improving laser line profile. A low coherence laser has been therefore used in the device: its power is 5 mW, which allows for a good signal to be acquired by the camera. However, this means a class II laser, which poses eye safety issues for the users and people nearby. The smart strategy to assure eye safety is briefly discussed at the end of Section 2.4.

**Figure 7:**
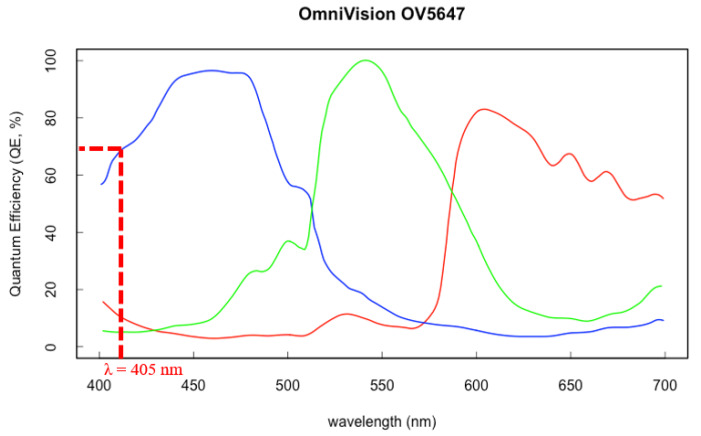
Spectral response of camera sensor’s selected [22].

As a consequence of the aforementioned aspects, the camera and its lens should have high sensitivity in the violet region, be compact, and provide an adequate resolution. Even if it is not mandatory in a triangulation system, the camera installed on the device discussed in this paper is an RGB camera: this choice enables the implementation of strategies for recognizing the surface color and adapting camera exposure. (discussed later in Section 2.4). The camera used in the device is a Raspberry PiCam (Raspberry Pi Foundation, Cambridge, UK). The camera uses a low size sensor (sensor optical size: ¼’’) with relatively high pixel distribution (2592 × 1944 pixels) adjustable focus (focal length: 3.6 mm; numerical aperture F#: 2.9) together with a good spectral response in the violet region (QE of 65% at 405 nm), as shown in Figure 7**.** Moreover, it is assembled on a small board (size: 25 × 24 × 9 mm;), which eases installation inside the cover of the smartphone.

### 2.2. Hand-Held Device: Software Design

The camera, operating at 8-bit, is driven by a Raspberry Pi Zero (Raspberry Pi Foundation, Cambridge, UK) installed in the casing. Once acquired, the RGB image is sent to the smartphone via type-C USB link. Then, image processing takes place through the computational resources of the smartphone. The smartphone communicates with the Raspberry Pi Zero exploiting the Termux environment (Android terminal emulator and Linux environment app), which is also used for running the image processing algorithms (Python) and the device’s human-machine interface (HMI).

When translating from 2D camera coordinates to 3D world coordinates, the accuracy of the estimated 3D coordinates of a point in the space is limited by the imaging system resolution. If the feature size targeted is beyond the resolution of the system, which is limited by the pixel size of the detector, algorithms which estimate features positions to subpixel accuracy are useful [23].

**Figure 8:**
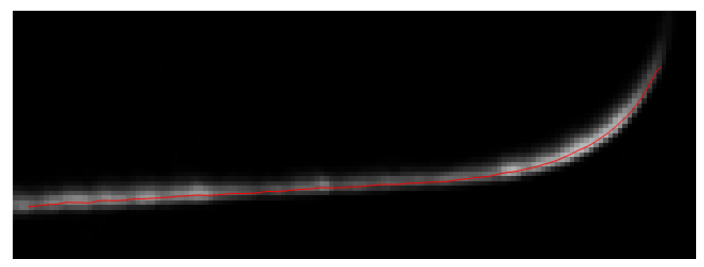
Line profile extraction with the Gaussian Kernel algorithm.

The image of the laser line projected onto the target surface is processed to determine its position with subpixel accuracy. The laser line runs almost horizontal in the acquired images and has a gaussian intensity profile; its vertical position is found by processing light intensity profiles along the columns, which are close to orthogonal to the line. This is done on the blue plane of the RGB image, since it is expected to be the one exhibiting the highest SNR, given the wavelength of the laser. The Gaussian Kernel algorithm [24], which balances performance and computational load, is exploited to extract the laser peak intensity. Figure 8 shows the laser line acquired by the blue channel of the camera and the superimposed red dots, representing the central intensity peak of the laser line, extracted by the Gaussian Kernel algorithm; the fitting is quite remarkable both in the straight part of the laser line and in the curved part.

**Figure 9:**
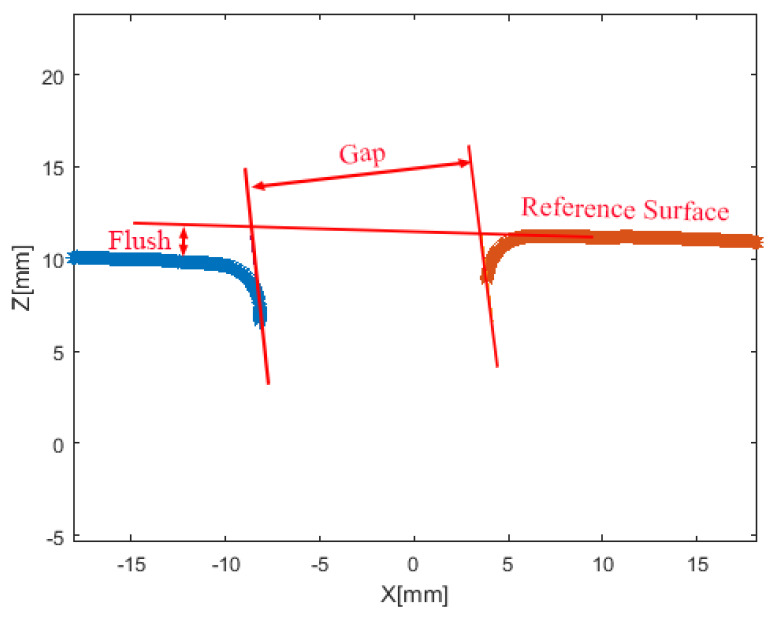
Gap and flush definition with the feeler/dial gauge algorithm.

Data points of the left and right edges are then clustered exploiting the k-means algorithm [25]. Results of this step are reported as red and blue lines in Figure 9. These point clouds are processed to extract gap and flush values.

The gap and flush algorithm simulates how a manual feeler gauge and dial gauge operate. The feeler gauge is a parallelepiped solid body inserted between the parts; its thickness is the measured gap. The dial gauge measures the flush as the distance of the tangent to one of the parts from the other part. Figure 9 describes the corresponding geometry, where the blue and red points are the result of the laser line profile extraction performed by the algorithm presented above. This choice in the evaluation of the gap was made to avoid the use of additional assumptions, e.g., bead radii, and to make it robust to geometrical changes on the profiles. The following steps are considered for extracting gap values:

1. Identification of the facing edges of the two clustered point clouds;
2. Identification of the two parallel lines tangent to both facing edges;
3. Calculation of the distance between the lines identified at the previous step;
4. For the flush determination the algorithm is based on the following steps:
  (a) Identification of a reference cluster among the two clustered point clouds (the reference coincides with a reference surface on the car, e.g., the frame when measuring gap and flush at the tailgate)
  (b) Identification of the straightest line fitting the reference point cloud;
  (c) Calculation of the distance between the line identified at the previous step and the other point cloud;
  (d) Recognition of the point cloud extremes;
  (e) Fitting the “reference” cloud with a straight line;
  (f) Determination of the angle corresponding to the maximum distance between the reference line and the other point cloud.

With respect to classical circle fit algorithms adopted in gap and flush extraction, the algorithm adopted shows higher robustness to noisy data and lack of data points on the groove.

### 2.3. System Calibration

Moving from pixel to real-world coordinates requires a calibration of the device. With respect to model-based approaches [26], a direct calibration [27] was considered. A direct calibration in a triangulation device consists in mapping the target distance z and the lateral position x to sensor coordinates (*i,j*). This can be done by traversing along the z direction a target made of parallel stripes orthogonal to the x direction. The calibration target is realized by a flat panel with equally spaced black and white parallel stripes. Stripes have been obtained by printing on a paper sheet on a flat rigid support at 1200 dpi. The uncertainty of the calibration target therefore is 0.02 mm for gap values in x and y direction. The uncertainty in z direction, corresponding to flush measurement, is 0.01 mm, limited by the micrometric traversing stage adopted.

During calibration, the smartphone is fixed on a support so that the laser line projects orthogonal to the target surface; alignment uncertainty is below 0.5°.

The calibration we performed maps real world *x* and *z* coordinates into pixel coordinates and uses a fitting second order polynomial model as the calibration model. This approach inherently accounts for all components of uncertainty, optical distortion, triangulation geometry, camera parameters.

**Figure 10:**
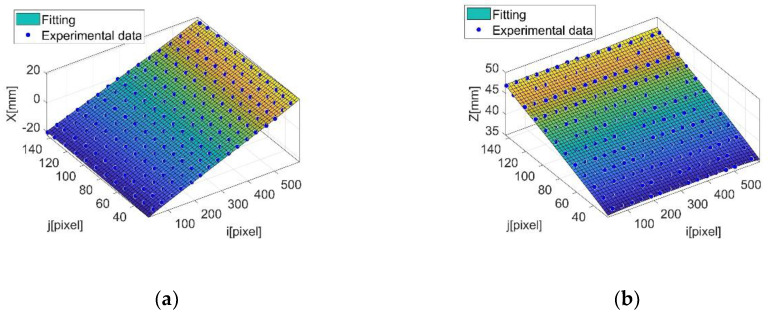
Point clouds x(*i*,*j*)—(**a**), z(*i*,*j*)—(**b**) and corresponding linear regression (calibration surfaces) *H_x_*(*i*,*j*) and *H_z_*(*i*,*j*).

The target is scanned over the z-range (or depth of field) of the device by a micrometric stage and for each z-position an image is acquired. Data obtained can be plotted as point clouds *x*(*i*,*j*) and *z*(*i*,*j*), *i,j* representing pixels, and then fitted with two polynomial surfaces *H_x_*(*i*,*j*) and *H_z_*(*i*,*j*), which will represent the calibration functions. Figure 10 reports the data clouds *x*(*i*,*j*) and *z*(*i*,*j*) acquired in the calibration process. superimposed to the polynomial fitting functions. The calibration uncertainty is then computed by estimating the standard deviation of the residuals of the experimental data with respect to the fitting function. The standard deviation of the residuals turns out to be 0.15 mm and 0.12 mm respectively for *x* and *z* surfaces. Then, the expanded uncertainty of calibration is *U_x_* = 0.30 mm in *x* direction (gap) and *U_z_* = 0.24 mm in *z* direction (flush) (coverage factor *k* = 2 and confidence level 95%) [19]. This is the calibration uncertainty of the device; in operating conditions uncertainty can increase, especially due to surface characteristics and to operator handling the device.

### 2.4. Smart Behaviors

As a hand-held device targeting HITL tasks, the device needs to ease interaction with the operator as well as ensuring that measurement performed by the operator can be trust (e.g., if the operator is asked to measure on a certain point—tailgate to body frame—the device should check whether she/he is measuring there and not somewhere else—e.g., headlights). Indeed, this is fully in line with the “Operator 4.0” vision addressed by Romero et al. [28]. A continuous interaction loop has been implemented: the device checks operator decisions by comparing them with its own strategies. If and only if results of these checks comply with pre-set acceptable strategies is measurement then allowed by the device.

**Figure 11:**
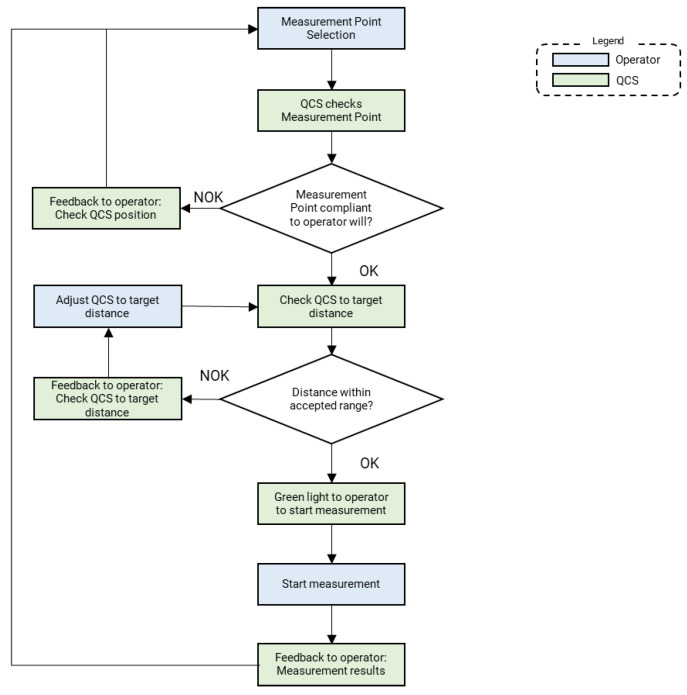
Quality control station (QCS) for gap and flush: measurement procedure.

The measurement procedure involves the operator at different levels, as shown in Figure 11. The operator interacts with the device through the touch screen of the smartphone, where a graphic interface (HMI) is displayed. At the beginning of the inspection, the operator is asked to select the measurement point and to point the device towards the measurement he/she selected. Then, to check operator decision and avoid him/her measuring in different points from the selection, a color picture of the part is automatically taken by the device, with the laser source switched off, and algorithms for recognizing and classifying the framed part are run. If the result of this classification states that the measurement point selected by the operator is really the one he/she pointed the device at, then the procedure can go on; otherwise, the operator is asked to check the right position of the device. This cross-check has a twofold implication: on the one hand, it makes it possible to reduce the assignation of gap and flush values to wrong measurement points, and on the other hand, it makes it possible to optimize the measurement conditions (i.e., optimize the exposure time of the camera with respect to materials/colors characterizing the part under inspection) to guarantee the largest possible contrast of the laser line image and, hence, a lower uncertainty on result of the measurement.

Indeed, since different materials/colors of the same car have different optical scattering characteristics at the same laser wavelength illumination, it is important to optimize the camera exposure time to guarantee the best image result. To perform this operation in an automated way it is fundamental to somehow recognize the measurement point on which the measurement is taking place. Indeed, once the measurement point is recognized, a recall of materials/colors characterizing that measurement point can be performed from a pre-set database and the best camera exposure time for that combination of materials/colors can be set.

The recognition of the measurement position is performed on the device by running a Convolutional Neural Network (CNN) named Alexnet [29]. This is a pre-trained CNN that takes the color image of the part (with laser source switched off) under inspection as input and classifies the image according to the classes defined during the learning phase (metal, plastic, glass, different color). Each class embeds an optimized exposure time determined during dedicated in-line experiments on different cars. Therefore, once a label is assigned to the part under inspection (classification result), the camera exposure time can be set accordingly, thus ensuring the best result in terms of image quality, laser line profile extraction, and gap and flush measurement.

**Figure 12:**
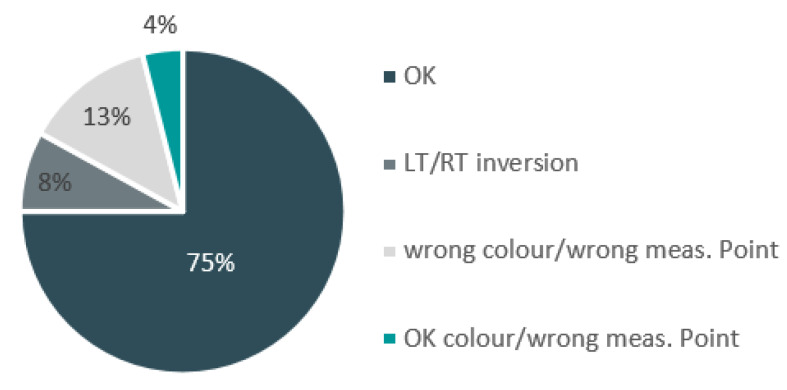
Convolutional Neural Network (CNN) classification results for labelling the part under inspection.

Figure 12 reports some results from a set of 62 images of different parts of the same car’s samples. The CNN dataset has been populated with 650 images of the same parts, with 30% of the images used for training and the remaining 70% for data validation. As can be seen, the CNN correctly classifies 75% of the images. The CNN fails in classifying the image received in the 13% of the whole population. Left to right inversion (LT/RT) represents the main source of classification error. This is quite straightforward, since the CNN is invariant to image scaling and rotation.

Given the few images that were used for training the network, these results are quite remarkable. Some misclassifications are still present. However, it is expected that, once the device runs continuously in the production line and more images are available, the behavior of the CNN becomes more and more stable, thus providing more accurate classification results.

Another smart behavior implemented in this hand-held triangulation system is to assure it is used within the calibration range of the device. For this purpose, an infrared time-of-flight distance sensor, mounted in the same enclosure hosting the laser triangulation, was embedded in the device with a twofold purpose—to enable the measurement to be consistent with the calibration range of the device and ensure the operator eye safety. In fact, the laser is switched on only if the measurement point is recognized as valid and the target-to-sensor distance is within the measuring range.

If and only if the results of these checks comply with operator decisions then the permission to start measurement is provided and a feedback to the operator when the measurement is done is given.

## 3. Results and Discussion

### 3.1. Laboratory Tests

To estimate the uncertainty of the measurement chain involving the device in operating conditions, a type-A uncertainty analysis according to the the ISO/IEC Guide 98-3:2008 (GUM) [19] has been performed. The procedure was run in a laboratory, on reference gaps and flushes. These were provided by a cubing car frame, a highly accurate benchmark for optimizing and qualifying assembly parts, realized by milling a full-size auto-body from solid aluminum; the reference car body is not painted. The light scattering of the surface is sufficiently good to assure a good signal-to-noise ratio in the acquired images. Therefore, this test allows us to estimate the uncertainty associated to the instrument when used by an operator in optimal optical conditions.

The cubing car frame has been used as a target for gap and flush measurement and cubing nominal values as nominal references. Nominal values have been verified by measuring the same points with calibrated pins at 0.01 mm uncertainty.

Gap and flush values have been measured with the device in 6 different points, for a total of 42 acquisition. Results have been statistically processed to derive expanded uncertainty. It results *U_x_* = 0.38 mm for gap and *U_z_* = 0.33 mm for flush values (95% confidence level). These results agree with the analysis performed during calibration; as expected, calibration uncertainty is slightly smaller than uncertainty observed in this Type A estimate on the cubing car used as reference. This can be explained by operator influence on the measurement. In particular, the operator, even if trained, is not capable of exactly aligning the laser line orthogonal to the gap and, moreover, lacks positioning repeatability.

### 3.2. In-Line Measurements

The hand-held device was finally tested in the production line of Volkswagen Autoeuropa (VWAE). The actual testing conditions, i.e., target in motion, cars of different colors, the dexterity of the operator handling the device, etc., may affect measurement uncertainty, which may significantly be larger than calibration uncertainty discussed above. Therefore, specific tests were performed to evaluate this in-use uncertainty. The same operator was asked to measure gap and flush, at the final check stage of the assembly line, on 28 different points of different fully assembled cars. This resulted in a total of 145 acquired data.

A manual caliper (0.05 mm uncertainty) is used as a state-of-the-art instrument in order to provide reference values of the gap.

**Figure 13:**
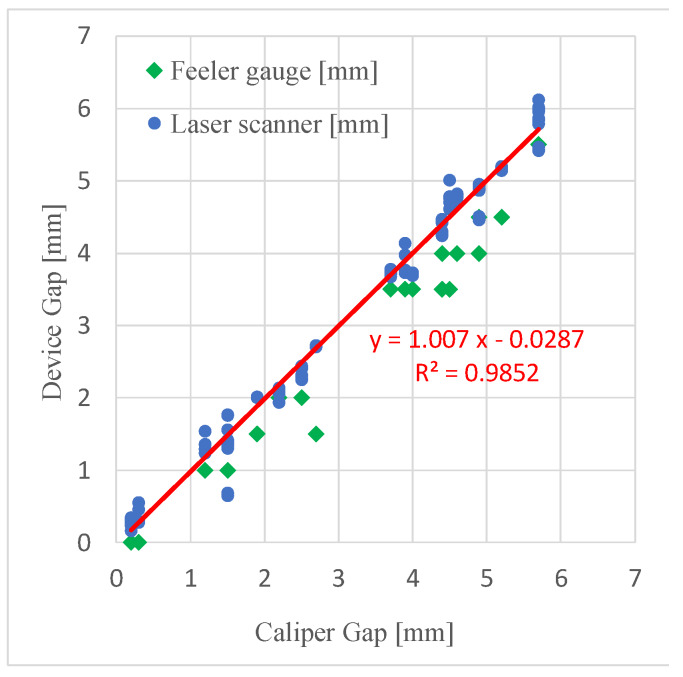
Comparison between the smart hand-held device and the feeler gauge measurement; data are plotted versus reference gap measured by a caliper.

Figure 13 shows the correlation between gap values measured by the smartphone device and the caliper; data are plotted versus the reference gap value. The data acquired by the laser device are the blue circles. The linear regression shows an excellent agreement of the device output to the reference gap measured by the caliper; in fact, we observe a linear fit of unitary slope, which confirms the accuracy of the device. The experimental data are dispersed around the regression line and the analysis of residuals allows to estimate uncertainty. The expanded uncertainty estimated according to the ISO/IEC Guide 98-3:2008 [19] is *U_x_* = 0.44 mm. This larger value, compared to uncertainty at calibration level (see Section 2.3) and to uncertainty in laboratory conditions (see Section 3.1), is mainly due to a combination of operator’s repeatability in placing the instrument in front of the measurement point on different points/cars, together with the varying optical characteristics and color of the different points/cars, which affect instrument performance in a real in-line application.

The gap values were also measured using the manual feeler gauge that is currently used in VWAE assembly line (green diamonds in Figure 13). From the data plot it is evident that the resolution of the feeler gauge, 0.5 mm, limits the accuracy; the gap measurement is systematically underestimated. This latter effect is due to the way a feeler gauge works; to be inserted into the gap, the size of the feeler gauge must be smaller/ equal to the gap value, thus determining a systematic offset to lower dimensional values. No measurements can exceed the gap value. The systematic error consists in an underestimate of about 0.34 mm, accompanied by a loss of sensitivity of about 4%. If we apply a linear regression to the feeler gauge data and then statistically process the residuals, we can estimate the measurement expanded uncertainty *U_x_* = 0.54 mm. This value, larger than the uncertainty of the smartphone device, is mainly due to the lack in resolution of a feeler gauge.

A similar analysis was conducted for flush, taking as a reference measurement by a dial gauge. The Type A uncertainty analysis, performed in the same way as above, provides an expanded uncertainty of *U_z_* = 0.58 mm for flush measurements performed by the laser device in a real production line on cars of different colors; again, this uncertainty is significantly larger than calibration uncertainty, due to the fact that the operator is not precise in handling the device and that the device itself operates in variable optical conditions on cars of different colors as well as on different environmental light conditions.

## 4. Conclusions

This paper has presented a smart hand-held device for gap and flush measurement to be used in the automotive assembly processes in compliance to the Industry 4.0 paradigms. The instrument hosts a laser line triangulation system integrated to a smartphone. The choice of a smartphone as a platform for the measurement system is due to the high computational power characterizing these devices, the presence of embedded sensors and cameras, to long-lasting batteries, the availability of a display and a touch screen, and their wireless connectivity capabilities. Indeed, a smartphone, when upgraded with proper sensing elements, is a fertile ground for building IIoT devices to be used in modern factories. The optical arrangement of the triangulation system is designed to minimize the overall size of the device. The system is designed to operate at a stand-off distance SO = 20 mm, over a range Δz = 15 mm, in order to make contact/non-contact measurements possible. The short-wavelength (λ = 405 nm) laser light adopted makes it possible to operate on smooth materials of different optical behaviors (reflective, transparent, translucent), still keeping an acceptable light scattering from the surface.

Gap and flush values are extracted by processing images at subpixel resolution by a Gaussian kernel algorithm and by running a dedicated algorithm tailored to reproduce caliper/dial gauge-based operations normally adopted by quality operators in the assembly line.

The uncertainty analysis performed showed an expanded uncertainty at calibration level of *U_x_* = 0.30 mm for gap and *U_z_* = 0.24 mm for flush; during calibration, the device is fixed on a rigid support and aligned orthogonal to the reference target. Uncertainty increases to *U_x_* = 0.38 mm for gap and *U_z_* = 0.33 mm for flush when an operator is taking measurements on a cubing car body, a precise geometric reference made in ground aluminum, not painted; this material provides rather good light scattering conditions; therefore, the instrument operates in optimal optical conditions. The increase of uncertainty is mainly due to operator dexterity, which is not capable of a precise alignment of the device orthogonal to the surface and to the gap. Finally, uncertainty increases to *U_x_* = 0.44 mm for gap and *U_z_* = 0.58 mm for flush, when taking measurements in operating conditions in a real assembly line, on cars of different color. This increase in uncertainty when moving from lab to the assembly line is mainly due to the operator repeatability and to the different optical conditions in which the instrument is operating.

The overall uncertainty observed in a real production environment is still within the specifications that a car manufacturer allows for the measurement of gap and flush during in-line assembly operations, therefore the device successfully fulfils its task.

Quality of the measurement is ensured by dedicated software strategies (e.g., automated measurement point recognition, adaptation of camera exposure time, etc.), while eye safety for the operator is ensured by the conditional switch on/off the laser source in accordance to a series of events (e.g., stand-off distance compliant to requirements). OPC-UA-based communication makes the device fully compliant to Industry 4.0 standards, which allows the device is to be considered as an enabler for the Operator 4.0, as it specifically targets HITL operations.

## 5. Patents

The device described in the present paper was patented as PCT/IB2019/051662, 2019.
